# Barriers and facilitators to using NHS Direct: a qualitative study of ‘users’ and ‘non-users’

**DOI:** 10.1186/s12913-014-0487-3

**Published:** 2014-10-25

**Authors:** Erica J Cook, Gurch Randhawa, Shirley Large, Andy Guppy, Angel M Chater, Nasreen Ali

**Affiliations:** Department of Psychology, University of Bedfordshire, Park Square, Luton, UK; Institute for Health Research, University of Bedfordshire, Putteridge Bury, Hitchin Road, Luton, UK; NHS England, Horley, UK; UCL School of Pharmacy, BMA House, Tavistock Square, London, UK

**Keywords:** Telephone-based healthcare, NHS Direct, Patients’ perspectives, Non-adoption, Barriers, Qualitative research

## Abstract

**Background:**

NHS Direct, introduced in 1998, has provided 24/7 telephone-based healthcare advice and information to the public in England and Wales. National studies have suggested variation in the uptake of this service amongst the UK’s diverse population. This study provides the first exploration of the barriers and facilitators that impact upon the uptake of this service from the perspectives of both ‘users’ and ‘non- users’.

**Methods:**

Focus groups were held with NHS Direct ‘users’ (N = 2) from Bedfordshire alongside ‘non-users’ from Manchester (N = 3) and Mendip, Somerset (N = 4). Each focus group had between five to eight participants. A total of eighty one people aged between 21 and 94 years old (M: 58.90, SD: 22.70) took part in this research. Each focus group discussion lasted approximately 90 minutes and was audiotape-recorded with participants’ permission. The recordings were transcribed verbatim. A framework approach was used to analyse the transcripts.

**Results:**

The findings from this research uncovered a range of barriers and facilitators that impact upon the uptake of NHS Direct. ‘Non-users’ were unaware of the range of services that NHS Direct provided. Furthermore, ‘non-users’ highlighted a preference for face-to face communication, identifying a lack of confidence in discussing healthcare over the telephone. This was particularly evident among older people with cognitive difficulties. The cost to telephone a ‘0845’ number from a mobile was also viewed to be a barrier to access NHS Direct, expressed more often by ‘non-users’ from deprived communities. NHS Direct ‘users’ identified that awareness, ease of use and convenience were facilitators which influenced their decision to use the service.

**Conclusions:**

An understanding of the barriers and facilitators which impact on the access and uptake of telephone-based healthcare is essential to move patients towards the self-care model. This research has highlighted the need for telephone-based healthcare services to increase public awareness; through the delivery of more targeted advertising to promote the service provision available.

**Electronic supplementary material:**

The online version of this article (doi:10.1186/s12913-014-0487-3) contains supplementary material, which is available to authorized users.

## Background

In 2012, NHS Direct was replaced with the new non-emergency ‘111’ telephone-based healthcare service. It was first introduced in 2010, followed by a national rollout in 2013. The aim was to provide a more integrated non-emergency service to provide a gateway for all non-urgent healthcare needs [1]. Although the introduction of ‘111’ has marked the end of NHS Direct [2], it has highlighted the increased role that telephone-based healthcare has within the NHS structure. Therefore, to understand patterns of NHS Direct uptake have provided an opportunity to learn valuable lessons about access and uptake of telephone healthcare based services. This knowledge can be applied to the ‘111’ telephone-based healthcare service, as well as services internationally, as countries worldwide adopt similar models of remote healthcare delivery [3-6].

NHS Direct provided 24 hour/7 day a week nurse led telephone-based healthcare advice and information to the public in England and Wales [7,8] (see Additional file 1). This service, introduced in 1998, marked a strategic shift towards the self-care movement [9] which encouraged the population to take an increased responsibility for their own health [8,10,11]. Evidence suggests that self-care is linked to improved health outcomes, improved quality of life, increased empowerment and patient satisfaction [11-13] and has been viewed as beneficial in reducing hospital admissions [14]. Consequently, self-care is now being viewed as an inextricable part of the individual care pathway, from maintaining a healthy lifestyle to caring for minor, acute and long-term health conditions [15].

NHS Direct has been at the leading edge of remote healthcare systems, directing healthcare into the 21st Century through the application of new technology solutions in primary care [16]. By 2011, NHS Direct received 8 million calls per year with reported high levels of satisfaction [17]. Whilst evidence suggests that there is an increasing shift towards self-care [11], with over 90% of people cited as being interested in taking more ownership of their health [18], the pattern is not uniform across all sections of society. For example, self-care uptake (and NHS Direct usage) has previously been reported to be substantially lower in those who are older (85+) [19], among the less affluent and deprived [20,21] and minority ethnic groups [22].

Uptake of telephone-based healthcare services has been explained by the technical performance and functional reliability of technology [23], concerns of personal privacy and security [22,24], money, perceived confidence to engage with health technology [20,25,26] and severity of health symptom(s) [25]. Perceived confidence to engage with health technology and severity of symptoms suggests that if an individual has low confidence to use health technology and has high perceived severity of illness, they are more likely to prefer face-to-face contact with a healthcare professional [26] and less likely to see the benefits in self-care [27]. Factors enabling self-care include awareness of the services, and service recommendation and signposting by healthcare professionals [18].

There is a dearth of evidence exploring explanations for usage and non-usage of NHS Direct. As the provision of healthcare moves away from face-to-face contact between patient and practitioner there is a pressing need to understand the reasons for usage and non-usage of telephone-based healthcare services to ensure that all sections of society are able to maximise opportunities for self-care. To examine the usage of NHS Direct this research makes a small, but valuable contribution, to help understand the barriers and facilitators to usage of telephone-based healthcare services.

## Methods

### Sampling and recruitment

Nine focus groups were conducted between October 2011 and January 2012. A trained researcher (EC) facilitated the focus groups with the support of a research assistant, both of whom had no direct connection with NHS Direct that ensured the focus groups were unbiased. Focus group methodology was used to generate data as it involves a group interaction which can help participants to explore and clarify their views in ways that may be less accessible in a one to one interview [28,29]. This methodology has been a commonly applied approach in health services research to identify views and attitudes towards health services [30-32].

Ethical approval was granted by the University of Bedfordshire ethics committee in March 2010 and the NHS Ethics Committee in April 2010 (REF: 11/H0301/8). All participants who took part in this study provided their written informed consent. Participants’ anonymity and confidentiality was ensured throughout.

Table 1 presents the demographic composition of all focus groups and shows that each focus group comprised of between five and twelve participants (a total of 81 participants: 62 females and 19 males). Participants’ ages ranged between 21 and 94 years with the majority White British. A purposive stratified sampling strategy [33] was used to recruit in three geographical areas in England to ascertain diversity of opinion.

**Table 1:** **Focus group composition and recruitment of NHS Direct users and non-users**

**Focus group**		**Location**	**Age**	**Gender**	**Ethnicity**	**Profile and characteristics**	**Focus group description**
1	Users (N = 8)	**Mid Bedfordshire**	21–46	Female (9)	White British (7) Mixed: Black Caribbean (1)	High geographical usage area – mothers with children (<5)	Participants recruited from Sure Start centres in Mid-Bedfordshire. Sure Start centres are open to parents, carers and children providing early learning and full day care for pre-school children.
2	Users (N = 9)	**Mid Bedfordshire**	23–54	Female (9)	White British (9)	High geographical usage area – mothers with children (<5)	Participants recruited from a range of Sure Start centres in Mid-Bedfordshire. Sure Start centres are open to all parents, carers and children providing early learning and full day care for pre-school children.
3	Non-users (N = 10)	**Mendip: Moor**	67–93	Male (6); Female (4)	White British (10)	Older residents with high levels of deprivation residing in isolated rural community	Focus groups were held as part of an existing community group which provides retired adults mainly older (65+) a range of social activities and events.
4	Non-users (N = 11)	**Mendip: Mells**	67–94	Female (11)	White British (11)	Older residents with high levels of deprivation residing in isolated rural community	Focus groups were held as part of an existing community group which provides retired adults mainly older (65+) a range of social activities and events.
5	Non-users (N = 9)	**Mendip: Creech**	64–92	Female (9)	White British (9)	Older residents living in larger isolated rural community.	Focus groups were held as part of an existing community group which provides retired adults mainly older (65+) a range of social activities and events.
6	Non-users (N = 11)	**Mendip: Beckington & Rode**	50–87	Male (3) Female (8)	White British (11)	Middle income families living in moderate suburban semis in a rural area.	Focus groups were held as part of an existing community group which provides retired adults mainly older (65+) a range of social activities and events.
7	Non-users (N = 7)	**Manchester: Baguley**	36–73	Male (2) Female (5)	White British (7)	Deprived ward resided by families in low rise social housing with high levels of benefit need.	Participants recruited from a range of community organisations which provide residents with their social, recreational and sporting needs.
8	Non-users (N = 11)	**Manchester: Gorton North**	16–84	Male (3) Female (8)	White British (11)	Deprived ward characterised by low income workers in urban terraces.	Participants were recruited from a range of community organisations which provide residents with their social, recreational and sporting needs.
9	Non-users (N = 6)	**Manchester: Longsight**	26–49	Male (6)	White British (2) Pakistani (2) Black African (2)	Deprived ward characterised by low income workers in urban terraces and culturally diverse areas.	Participants were recruited from a drop in community centre which provides residents a range of activities focusing on improving health and wellbeing.

### NHS Direct ‘users’

NHS Direct ‘users’ were purposefully chosen as mothers with young children (<5 years). Research suggests that this population group accounted for over 20% of all calls made [21,24] and represents the highest ‘users’ of NHS Direct [21]. NHS Direct ‘users’ were recruited through two Children’s Activity Centres in Mid-Bedfordshire, as these sites were based in high geographical usage areas [24]. Prospective participants were approached by the lead researcher (EC) and invited to take part. If they were interested they were then screened to ensure that they met the inclusion criteria.

The inclusion criteria outlined that prospective participants were a mother of a child (<5 years) and had used NHS Direct at least once in the previous year for either themselves or their child. A total of two focus groups were held within this sample group before saturation was achieved [34]. Participants (N = 17) were aged between 21 and 54 (M = 32.59; SD = 8.4), the majority classified themselves as White British (N = 16), with one participant who identified herself as Mixed White and Black Caribbean (Table 1).

### Non NHS Direct ‘users’

Two Local Authorities were chosen, one urban and one rural with mortality used as a proxy to identify need. This approach allowed for the identification of geographical differences of life expectancy between regions, districts, wards and output areas [35,36]. Though the usage of expected life expectancy birth data, Local Authorities were chosen as (1) lowest life expectancy urban local authority area defined as urban 1 (predominantly major urban), and (2) the lowest life expectancy rural local authority area defined as urban 6 (predominantly rural 50/80) [37].

Manchester was the chosen urban local authority which has the lowest life expectancy from birth which currently stands at 72.5 (CI: 72.1–72.8). Mendip, located in the South West of England was the rural local authority chosen with the lowest life expectancy from birth which stands at 77.5 (CI: 76.8–78.2) [38]. Both geographical areas suffer from higher than average levels of deprivation. Manchester is ranked the fourth most deprived local authority in England [39], whilst Mendip is shown to have high levels of unemployment with pockets of deprivation throughout [40].

**Figure 1:**
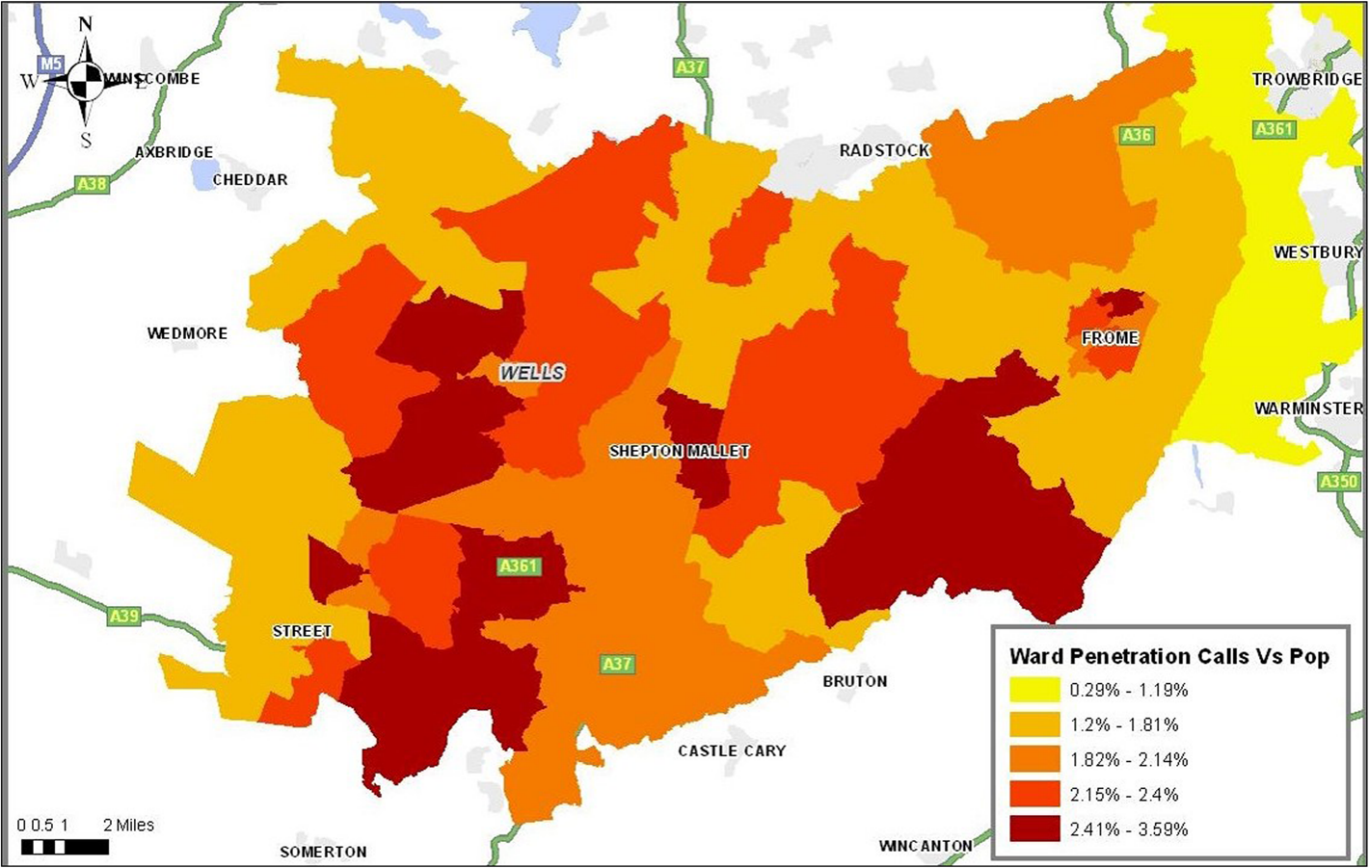
**Penetration of calls to Mendip at ward area.**

**Figure 2:**
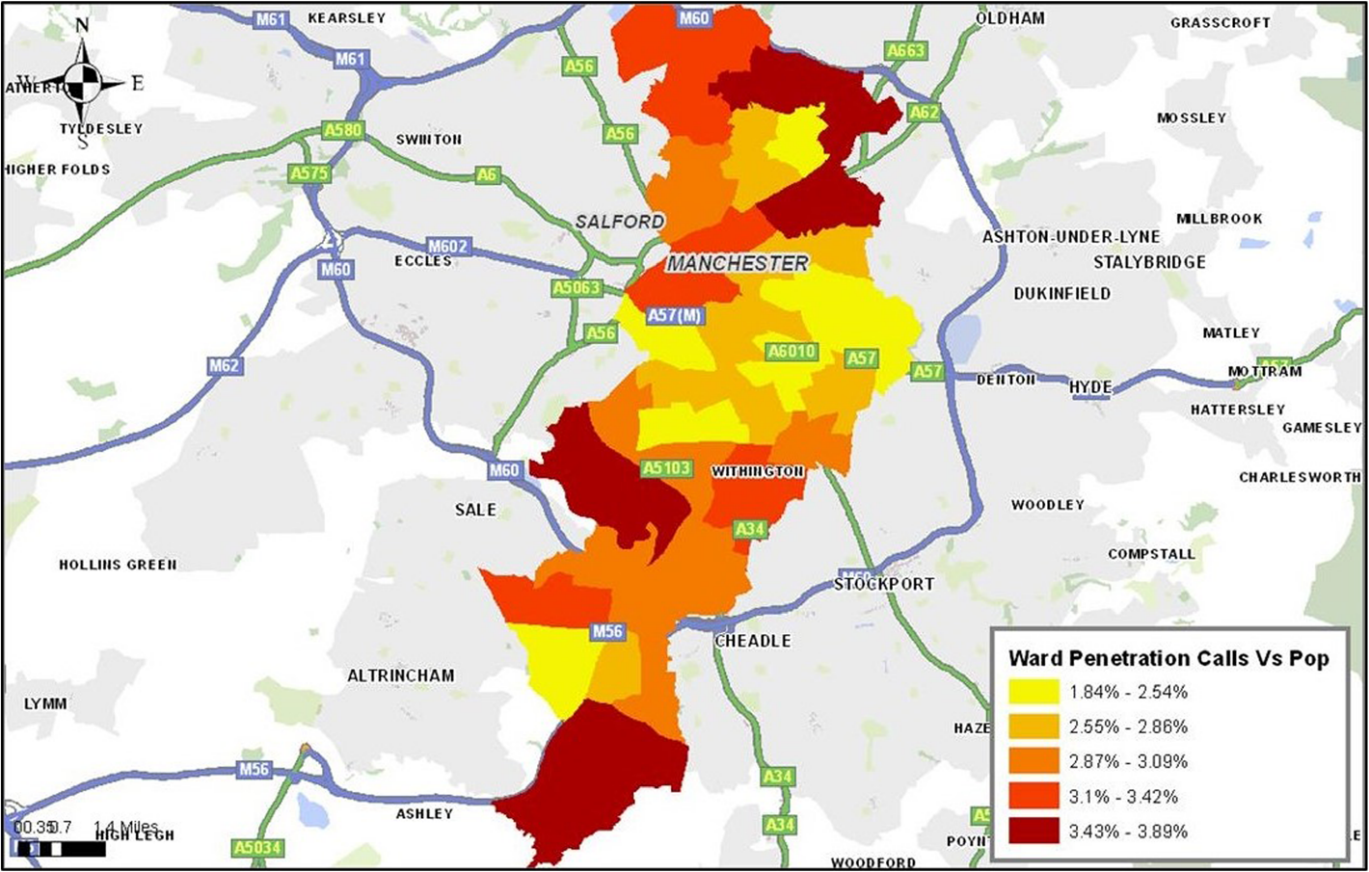
**Penetration of calls to Manchester at ward area**.

A stratified ‘stratum’ sampling approach was then used on the basis of low geographical usage at ward level which was carried out and mapped NHS Direct call data and compared this to the concentration of calls by population through the use of geographical information system software ArcGIS [41] (Figures 1 and 2). The lowest usage wards were then explored using population segmentation (Mosaic) which provided detailed information that defined the population subgroups by a mix of demographic, cultural, behavioural, psychosocial, geographic factors [42] (Table 1). In relation to demography they should meet the characteristics of ‘non-users’ of NHS Direct depending on the ward chosen.

### Mendip focus groups

A total of four focus groups were carried out in Mendip in the wards Moor, Mells, Creech and Beckington & Rode. The Mendip sample were screened for age and were required to be ≤50 years as this population sub-group represent the lowest users of NHS Direct [24,43,44]. This sample (N = 41) were predominantly White British females with ages ranging from 50–94 (M = 79.93, SD = 10.08) (Table 1).

### Manchester focus groups

A further three focus groups were organised with ‘non-users’ in Manchester in the wards Gorton North, Longsight and Baguely. The Manchester sample (N = 24) were also screened for representation of the geographical area and aimed to capture cultural diversity (Longsight) deprivation (Gorton North, Longsight and Baguely) and variation in gender within the residing wards (Table 1).

### Setting

All community centres and day centres in the ward areas were visited by the lead researcher (EC) in person. The centre manager was provided with a recruitment poster, a lay overview of the study, alongside a participant information sheet which detailed the study and outlined the inclusion/exclusion criteria. This information was then disseminated to prospective participants and those who were interested were asked to provide their name and availability to the centre manager.

The focus groups were held in community or day centres. In Mendip some of the focus groups were held as part of an existing group; for Manchester, the focus groups were not part of an existing group. However, all participants were familiar with each other which allowed them to feel at ease within a familiar setting. Before the focus group took place, all participants were screened by a researcher (EC) to ensure that they met the inclusion criteria i.e. they reside in the ward defined and have not used NHS Direct service for either themselves or another person. Each focus group session lasted approximately 90 minutes and was audiotape-recorded with participants’ permission.

### Focus group process

Participants were asked questions surrounding their awareness of NHS Direct, why they have or have not used the service, the advantages and disadvantages around using this service, structural and perceived barriers relating to positive and negative attitudes. Questions also centred on the usefulness of the service and ease of use, alongside their attitudes towards communicating with healthcare professionals via the telephone. At the end of each focus group, the researcher gave the participants an opportunity to comment on the data and on the key themes that had emerged from the discussion to check and confirm accuracy. No factual errors were found in the data and there were no requests for amendments or amplifications. Data collection stopped once saturation had been reached.

### Analysis

Focus groups were audiotape-recorded and transcribed verbatim using pseudonyms by a member of the research team (EC). The framework approach [45] was then used to thematically analyse the data which provides a generative analytical procedure that uses distinct connected stages of coding allowing for cases to be compared [46]. The analysis closely followed the five distinct stages of analysis; (1) familiarisation, whereby the researchers independently reviewed a sample of the transcripts; (2) identify a thematic framework, whereby two researchers (EC & NA) independently identified and organised key themes after coding the first few transcripts and cross checked. All major themes achieved consensus. The next stage (3) indexing, whereby the lead researcher (EC) independently applied the themes to the text. This was followed by stage (4), charting, where the data was managed and summarised using Excel whereby the summaries for each code were transferred into cells with assigned page numbers to track the narrative. The final stage (5), mapping and interpretation, occurred through exploration of the relationships and patterns within the data [47], and was completed after research team discussions.

## Results

Five themes emerged throughout the analysis of the transcripts which related to awareness of the service, costs to the individual, ease of use, time/speed, and acceptability of non-face-to-face healthcare. Similarities and differences between ‘users’ and ‘non-users’ are identified for these themes where relevant (Table 2).

**Table 2:** **Overview of similarities and differences of barriers/facilitators across the sample groups towards using NHS Direct**

**Theme**	**User groups Bedfordshire**	**Non-user groups Manchester**	**Non-user groups Mendip**
**Awareness of service**	• Good awareness and understanding of service	• Lack of awareness	• Lack of awareness
	• Most participants had used a wide range of services NHS Direct had e.g. online self-assessment tool	• Most participants had not heard of NHS Direct or services they provide	• Most participants had not heard of NHS Direct or services they provide
		• Some misunderstandings of what NHS Direct is	• Some misunderstandings of what NHS Direct is
**Cost to the individual**	• Most participants were not aware of the cost from a mobile phone	• Viewed as very expensive	• Expense was not viewed as a barrier
	• All participants had a landline phone	• Many of the participants did not have a landline phone	• All participants had a landline phone
**Ease of use**	• All participants found the service easy to use	• Some participants felt that this would be an easy to use service	• Difficulties in hearing over the phone
	• Viewed easier than using conventional out-of hours services	• Concern of complicated phone service with lots of options	• Dislike of answering lots of questions over phone
		• Being passed from person to person	• Difficulty of understanding foreign accents
		• Language barriers e.g. non English speaking	• Technical issues e.g. afraid of being cut off
			• Memory would make it difficult to use
**Time/Speed**	• Seen as instant advice and reassurance	• Concerned about waiting a long time for a call back	• Concerned about waiting a long time for a call back
	• Was viewed as a key advantage to using the service	• Was viewed as wasting time	• Was viewed as wasting time
	• Sometimes there was a long time to wait for a call back from a nurse		
**Acceptability of non-face-to-face healthcare**	• Positive attitudes towards not having face-to-face contact	• Preference for face-to-face healthcare	• Preference for face-to-face healthcare
	• Provided reassurance	• Would feel that they are unable to express themselves	• Would feel that they are unable to express themselves
	• Viewed service as personable and professional	• Would not provide reassurance	• Would not provide reassurance
		• Was not viewed as personable	• Was not viewed as personable

### Awareness of service

Overall, NHS Direct users had a good awareness and understanding of the service. They were aware of all individual services on offer including the core triage provision, health information and medicine advice services. Many participants were also aware of the internet based services, including the health encyclopedia and the Self-Assessment Tool software, which many had used to receive a call back relating to symptoms either for themselves or their children. There was a variety of ways in which the participants had heard about NHS Direct. Many ‘users’ were directed to NHS Direct through their GP answer phone machine when they had phoned their surgery out of hours.

*‘When I first called it I had called my doctor and the doctors surgery didn’t have an out of hours so they actually give you the NHS Direct number so that’s how I knew the number’ (NHS Direct ‘user’, FG1)*

However others were made aware of NHS Direct through their midwives when they had children.

*‘I think it was from the midwife when I had just given birth, she came to the house to do a check and she gave me the number then’ (NHS Direct ‘user’, FG1)*

One participant saw the service advertised through yellow pages (a telephone directory), and also recalled seeing through local level advertising. In fact, a number of participants recalled a small credit card leaflet which had the telephone number on which participants could keep in their wallet.

*‘I think I knew through getting information through the post….it was a white card with blue writing’ (NHS Direct ‘user’, FG2)*

Conversely, in Manchester and Mendip there was a distinct lack of awareness was evident across all ‘non-user’ focus groups. Many of the participants had never heard about NHS Direct or the services that they provide. There were also uncertainties and misunderstandings of what services NHS Direct offered. For example, a number of participants thought that NHS Direct was a walk in clinic or provided an out of hours GP service.

*‘I’ve heard about it it’s supposed to make life easier or that’s all I have heard it supposed to do with phone calls or Internet and that’s about it’ (NHS Direct ‘non-user’, FG7)*

*‘I think some people myself included are getting confused with people ringing NHS Direct with people who ring their out of hours duty officer’ (NHS Direct ‘non-user’, FG6)*

### Costs to the individual

NHS Direct operated from a ‘0845’ number, which is a cost of a local rate from a landline. However, the cost is substantially higher from a mobile phone when not covered by an inclusive minutes plan [48]. It is important to note that the researcher did not explain the cost to identify awareness of this, so anything relating to cost was brought up by the participants.

Amongst the NHS Direct ‘users’ only one participant mentioned the cost of the phone call, whereby she spoke of her friend who was a single parent and could not access the service because of the expense incurred on the use of her mobile phone. Many of the ‘users’, use landlines to phone NHS Direct and were not aware of the cost implications to use a mobile phone. However, when they realised this all participants said that this would not affect future usage.

*‘She’s a single parent and she’s only got her mobile phone and she said the only issue she has because it’s an 0845 number and on her mobile it costs a lot….because she only has her mobile its three, four, five pounds’ (NHS Direct ‘user’, FG1)*

However, ‘non-users’ in the focus groups in Manchester were much more aware of the cost incurred when using NHS Direct, whereby this service was viewed as very expensive. Many of the participants did not have landline phones so had to rely on using mobile phones to access the service.

*‘The cost is a big issue especially if you don’t have a landline and if you have to do on a mobile phone if you are on pay-as-you-go then contract it’s dearer’ (NHS Direct ‘non-user’, FG7)*

*‘It is a paid number it puts people off that it isn’t a free number we only get credit once a fortnight when we get paid on our phones it’s true we can’t phone up no one here has landline phones’ (NHS Direct ‘non-user’ FG8)*

*‘The area that we live a lot of people who do have mobile phones that are pay-as-you-go, and it’s an extortionate amount that it costs on the phone. By the time you have got through your credit could go halfway through or even run out’ (NHS Direct ‘non-user’, FG7)*

Participants felt that if something was seriously wrong they would just phone ‘999’ (emergency phone line in the UK) as this was a free number. The ‘non-users’ felt that if NHS Direct was free to access they would be more likely to use the service. Although, there were discussions of concern that surrounded how the money to cover the cost of the call would be subsidised and if this would subsequently lead to further cuts to local NHS health services.

*‘If you are really poorly and you have a mobile phone and you have no credit on there then you can’t ring NHS Direct but you can ring 999 and get an ambulance to you for free’ (NHS Direct ‘non-user’, FG7)*

In contrast, the majority of the Mendip participants did not mention the cost of the telephone call throughout any of the focus group session. However, many did not use mobile phones and they all had access to a landline phone. At the end of the focus group the researcher explained that the calls are charged at a national rate and the cost may be substantially higher when using a mobile phone, but no participants advised that this would impact on their decision to use this service.

*‘Well not if it’s an emergency you would just pay it’ (NHS Direct ‘non-user’, FG6)*

### Ease of use

All of the participants who had used NHS Direct found the service easy to use with many participants highlighting that it was easier to use than using conventional out of hour’s services e.g. GP co-operatives, Accident and Emergency, pharmacies. The main benefit disclosed was that you would not have to leave the house.

*‘You don’t have to go through the process of packing and putting everyone in your car. You don’t have to leave all the children with such and such the ability to have to deal with the problem without having to up sticks also if you are on your own. If you feel rubbish you wouldn’t get in the car and drive’ (NHS Direct ‘user’, FG2)*

However, for participants in the Manchester sample there was a mixed response. Whilst there were a few participants who felt that they would find NHS Direct easy to use, the majority felt that to use the telephone would involve many deterring issues. For example, there was a perception through prior experiences of use of telephone services that there were too many options which would make it more complicated to use.

*‘It’s supposed to make life easier but I spoke to a friend of mine who has used it because she’s a mum and she had to press that many options that she found it easier to get the doctors to come out than use NHS Direct (NHS Direct ‘non-user’, FG7)*

Another perceived barrier which would impact on the ease of use, was the belief of being passed from person to person, which was felt as frustrating and would increase anxiety, especially when the call relates to an individual’s health. There were a number of issues about speaking to somebody on the telephone as opposed to face-to-face. For example, one non-user was dyslexic and stated that he finds it easier to speak to his GP face-to-face due to the difficulties to express himself.

*‘I’m dyslexic so it is better to see a doctor if I am ill so we can understand each other’ (NHS Direct ‘non-user’, FG9)*

Another issue related to language barriers. For example, not speaking English fluently was felt to impact negatively upon ease of use and confidence of using the service. The researcher did explain that NHS Direct did operate a translation service ‘language line’. However, none of the participants were aware that this service existed.

*‘Some people might not be able to call NHS Direct because some people can’t speak English or their English isn’t very good especially if someone is living on their own and their English isn’t good or there’s been very little English obviously they won’t feel confident’ (NHS Direct ‘non-user’, FG9)*

Particularly for the Mendip sample, there were a range of barriers that would impact on ease of use. The biggest concern related to hearing, where many of the participants relied on using their hearing aids that made it difficult to communicate over the telephone. They felt that this would prove difficult when they have to explain symptoms when they could not hear what was being asked of them.

*‘Relies on the person giving the call giving accurate description of their symptoms so they’re trying to explain how they feel and your elderly you can’t hear very well and you’re stressed and you’re on your own it’s not an ideal situation’ (NHS Direct ‘non-user’, FG5)*

Hearing was also a concern in relation to whom they would speak to. Participants from Mendip highlighted that they found foreign accents difficult to understand on the phone and often had to ask them to repeat themselves which they felt would prove difficult.

*‘I know there have been instances where you have been confronted by an Asian voice which is incredibly difficult to understand what she was saying which can be a massive language barrier’ (NHS Direct ‘non-user’, FG4)*

Participants from Mendip also discussed technical issues. For example, one participant from Creech, stated that there are a lot of technical issues related to the use of the telephone such as being cut off.

*‘In my opinion there is a lot of technical issues with the phone for example the line went dead so what do you do in that situation’ (NHS Direct ‘non-user’, FG5)*

Other physiological barriers related to memory, which was also suggested to impact on the ease of use.

*‘People with memory problems wouldn’t be able to think or remember what to do, where to get the number etc.’ (NHS Direct ‘non-user’, FG6)*

### Time/speed

For NHS Direct ‘users’, speed to obtain healthcare advice was the key advantage of the service, whereby the majority of participants viewed this service to provide ‘instant advice and reassurance’, and valued being able to speak to a trained nurse or healthcare professional quickly.

*‘They give you immediate feedback on what you need to do when you are in that situation’ (NHS Direct ‘user’, FG2)*

However, some NHS Direct ‘users’ did not agree with this perspective, and had some negative experiences that related to the amount of time it took to be called back by a nurse, and the time of day that they were called back e.g. being called during the middle of the night. For some participants, to wait a long time was perceived as reassurance, as it reflected that they were considered to be a low priority in terms of concern for their health condition.

*‘Apart from sometimes NHS Direct have taken 8 hours to phone me back I could have had an appointment in that time’ (NHS Direct ‘user’, FG2)*

NHS Direct ‘non-users’ from Manchester and Mendip felt that waiting was a core barrier to use the service, whereby there was a distinct preference for instant face-to-face healthcare. Many of the participants shared concerns about the wait to be called back and did not like the thought of to wait on the telephone for long periods. There was a perception that NHS Direct was seen as a side step of out-of-hours care so was seen as ‘wasting time’.

*‘I know a young carer she’s 24 looking after her mum with dementia who has seizures and every time she has got through (to NHS Direct) she has said it has been quicker to find a doctor and the doctors come out quicker than that because when her mum is bad she can’t be spending 10 min on the phone’ (NHS Direct ‘non-user’, FG8)*

However in contrast, two ‘non-users’, from Longsight, Manchester, felt that NHS Direct could save time to provide instant reassurance instead of going straight to an Accident and Emergency Department in a hospital.

*‘Accident and emergency is reduced (and you) save time’ (NHS Direct ‘non-user’, FG9)*

*‘NHS Direct is more instant if a person does have a problem’ (NHS Direct ‘non-user’, FG9)*

### Communication and non-face-to-face healthcare

NHS Direct ‘users’ felt the service gave them reassurance and enabled them to make the decision whether to escalate their health concerns or not. They also felt it gave them the reassurance that they had sought advice from a trained healthcare professional. None of the NHS Direct ‘users’ were concerned that it was not a face-to-face service. In fact, many ‘users’ highlighted that they preferred the lack of face-to-face contact, and viewed the service as both personable and professional which provided them with the level of reassurance they needed.

*‘I think the relief that it gives you in order to have someone to speak to and that you have actually looked into it. It’s now like you can now get on and follow the guidance but knowing that it is the trained nurse that phones you back is just useful’ (NHS Direct ‘user’ FG1)*

Conversely, ‘non-users’ from both Manchester and Mendip outlined an overarching preference for face-to-face healthcare. ‘Non-users’ felt that face-to-face healthcare offered more reassurance than speaking to somebody on the telephone. They also felt that if it was face-to-face they would be able to express themselves better and would feel more at ease to ask questions.

*‘If you felt that you needed reassurance you just take your children or yourself to hospital at least that way they can see you face-to-face or get the paramedic out then they would make that decision if you need to go to hospital…..to be honest face-to-face is really important because this is what reassures you and this has to be the best option’ (NHS Direct ‘non-user’, FG7)*

*‘You can’t talk about that you have got a high pressure you can’t do that over the phone…often physical symptoms are important aren’t they so I think it’s very necessary to see a doctor face-to-face’ (NHS Direct ‘non-user’, FG4)*

There were strong positive attitudes towards face-to-face communication. It was felt important by ‘non-users’ that an individual could express themselves through body language. It was also more personable when speaking to someone face-to-face. Participants agreed that personal face-to-face interaction with a healthcare professional is an integral aspect when seeking healthcare advice, which presented a barrier to using telephone-based health services such as NHS Direct.

*‘Seeing someone in person is friendlier like if you went to see someone and you talk to them you can see them and see them smiling at you and treated sympathetically but on the phone it’s different you don’t see….I just think it is more personal rather than the telephone’ (NHS Direct ‘non-user’, FG9)*

## Discussion

This study has explored the barriers and facilitators to use NHS Direct, a hitherto under researched area. This research has uncovered explanations for usage and non-usage of NHS Direct. The core themes which emerged from the focus group discussions were related to awareness, costs to the individual, time/speed of the service and the acceptability of non-face-to-face communication. This research highlights that participants’ views on self-care varies by age, ethnicity and socio-demographic factors [16].

NHS Direct ‘users’ held a good awareness of all services that NHS Direct provide. However, there was a distinct lack of awareness among the ‘non-users’. Whilst many individuals from both Mendip and Manchester had heard of NHS Direct through media and out-of-hours signposting, there was a clear misunderstanding, with many who believed that it was a walk in service that operates out-of-hours. This supports research that has suggested awareness of this service is low [43,49] which indicates that the impact of previous advertising campaigns has been largely unsuccessful in reaching all sections of the population. It is clear that awareness is a core mechanism which impacts on health service uptake [50] and, therefore, these findings reinforce the importance to provide clear information through tailored promotional campaigns to ensure all sections of the population are informed.

NHS Direct ‘users’ suggested that they did not view the cost to use NHS Direct was a barrier, with many not aware of the cost implications to use the service. Conversely, ‘non-users’ from Manchester felt the service was extremely costly, especially as many relied on pay as you go mobile phones. This view was not reported by ‘non-users’ from Mendip, which suggests that the cost of the service appears to be an access barrier for those in deprived communities who are unable to afford to use the service. As such, it appears that NHS Direct and other telephone-based services should be aware of the impact that cost may have on uptake by individuals from more deprived communities. Nonetheless, as the new non-emergency ‘111’ NHS phone line is rolled out nationally as a free service it will become even more important to communicate to the public that service has no cost, so this should not be a barrier to access.

A particular advantage of NHS Direct for ‘users’ was that the service was accessible and easy to use. However, the predominantly older Mendip sample felt that there would be issues that relate to hearing and memory that would impact on discussing healthcare information via the telephone. Older peoples’ access to modern technology has been extensively debated with research that suggests that, not only physiological changes associated with ageing such as decrements of sight, hearing, dexterity, motor functioning, co-orientation and cognitive processing can impact on newer models of healthcare [51-53], but also a wide range of psycho-social factors. For example, uptake has been strongly dependent on income, education, experiences, and attitudes [54], with confidence that relates to ease of use, shown to influence significantly older people’s adoption and use of new technology [54-56]. There is an assumption that there should be a ‘universal’ take-up of technology [57,58]. Whilst this assumption is challenged [59], access to technology driven healthcare can be increased through two main ways: (1) ensure that the service is easy to use, and (2) through the provision of tailored information to enhance awareness of such services within the UK’s diverse population.

A key advantage for ‘users’ was that NHS Direct was a quick way to access advice and health information. However, ‘non-users’ discussed the preference for ‘instant’ face-to-face reassurance with NHS Direct viewed as a diversion. On the other hand, ‘non-users’ suggested a clear preference for more traditional face-to-face health services both in and out-of-hours. This appears to support previous literature that has identified that older people [30,60], ethnic minority groups [32,61] alongside those from socially deprived communities [62], prefer and have more confidence with face-to-face healthcare communication. This could also relate to the fact that ethnic minority groups [63], older people and those who are from more deprived backgrounds prefer doctor-centred healthcare [64,65] and prefer to take a passive role in their health.

Whilst this research provided a wide overview of the facilitators and barriers of a telephone-based healthcare service there were some limitations that are noteworthy. Firstly, the NHS Direct ‘users’ focus groups only focused on one high ‘user’ group i.e. females with young children. This was also reflected by an imbalance between the numbers of participants in the ‘user’ versus ‘non-user’ focus groups (17 v 54). This imbalance is an outcome of the breadth of issues uncovered in the ‘non-user’ focus groups alongside the inclusion of ‘user’ focus groups which captured a diversity of opinion through a wide range of geographical and socio-cultural factors. Nonetheless, focus groups from other ‘user’ groups, such as younger adults aged 20–34, may have provided further insight into the barriers and facilitators of such health services.

Secondly, whilst there was an attempt to capture ethnic diversity, this was only evident in one focus group. As such, future research should aim to examine the barriers and facilitators of such services accounting for a wider variation of ethnicity. In particular, studies should focus on other ‘non-users’ (e.g. Eastern European, Chinese and Black African [24]) to determine the range of cultural factors that impact on the engagement of telephone-based healthcare. Finally, some of the focus groups were existing groups, in particular the Mendip sample. There were clear challenges to recruit older participants, and whilst this may have created some bias, it showed to be a useful way to reach a ‘hard to reach’ community sample.

## Conclusions

This research uncovered a wide range of factors which impact on the uptake of NHS Direct. Acceptability of non-face-to-face healthcare was a key driver to use NHS Direct. Whilst ‘users’ found the service both convenient and easy to use, ‘non-users’ emphasised a clear preference for face-to-face healthcare. This was supported by a lack of confidence in discussing healthcare over the telephone, particularly in older groups who had cognitive and sensory difficulties. Awareness and cost also impacted on usage, whereby ‘users’ showed a higher level of knowledge and awareness of the service. Conversely, ‘non-users’ had a low awareness and the cost of phoning a premium ‘0845’ number was also viewed as a barrier, particularly from those in deprived communities who rely on a mobile phone.

It is apparent that although some barriers are the same for both groups of ‘non-users’ in Mendip and Manchester, there are some differences. This suggests that a one size fits all approach cannot be adopted. Instead socio-demographic factors need to be taken into account to identify the barriers to enable the service to become more accessible to all communities. Therefore, if other similar services such as the new ‘111’ service are to become a more widely used model of remote healthcare then it is essential that the barriers and facilitators to access telephone-based services are addressed. Increased access will subsequently improve the patient experience and the urgent care pathway. In turn this will reduce the need of unnecessary visits to already overstretched healthcare services. A recognition of the factors that do and do not make people access and use services such as NHS Direct, will help to mobilise patients towards the self-care model and support them in to take responsibility for their own care [3,11,12].

## Additional file

Additional file 1:
**An overview of NHS Direct.**

## References

[1] **The New Number for the Future of Non-Emergency Health Services** [http://webarchive.nationalarchives.gov.uk/+/www.dh.gov.uk/en/MediaCentre/Pressreleases/DH_118861]

[2] Calkin S (2013). NHS Direct to Close at the End of the Financial Year.

[3] College of Nurses of Ontario (2009). Practice Guideline: Telepractice.

[4] Stacey D, Noorani HZ, Fisher A, Robinson D, Joyce J, Pong RW (2003). Telephone Triage Services: Systematic Review and a Survey of Canadian Call Centre Programs.

[5] Roland M (2002). Nurse-led telephone advice. Med J Aust.

[6] Goodwin S (2007). Telephone nursing: an emerging practice area. Nurs Leadersh.

[7] Department of Health (1997). A Modern and Dependable NHS.

[8] Department of Health (2000). A Plan for Investment- A Plan for Reform.

[9] Beveridge W (1942). Social Insurance and Allied Services.

[10] Wanless D (2002). Securing our Future: Taking a Long-Term View - the Wanless Report.

[11] Department of Health (2005). Self Care - A Real Choice.

[12] O’Cathain A, Goode J, Luff D, Strangleman T, Hanlon G, Greatbatch D (2005). Does NHS Direct empower patients?. Soc Sci Med.

[13] Bodenheimer T, Lorig K, Holman H, Grumbach K (2002). Patient self-management of chronic disease in primary care. JAMA.

[14] Purdy S (2010). Avoiding Hospital Admissions: What Does the Research Evidence Say?.

[15] Department of Health (2005). Self Care Support: Baseline Study of Activity and Development in Self Care Support in PCT’s and Local Areas.

[16] Mark AL, Shepherd IDH (2007). Leadership in a Leading Healthcare Organisation - the Development of NHS Direct West London.

[17] **NHS Direct** [http://www.politics.co.uk/reference/nhs-direct]

[18] Department of Health (2005). Public Attitudes to Self Care Baseline Survey.

[19] Hsu W, Bath PA, Large S, Williams S (2011). Older people’s use of NHS Direct. Age Ageing.

[20] Cook E, Randhawa G, Large S, Guppy A, Chater A (2013). Who uses telephone based helplines? Relating deprivation indices to users of NHS Direct. Health Policy Technol.

[21] Cook E, Randhawa G, Large S, Guppy A, Chater A (2012). A UK case study of who uses NHS Direct? Investigating the impact of age, gender and deprivation on the utilisation of NHS Direct. Telemed J E Health.

[22] Bibi M, Attwell RW, Fairhurst RJ, Powell SC (2008). The usage of NHS Direct by different ethnic and gender groups in an urban population. J Chronic Dis.

[23] Davis FD (1993). User acceptance of information technology: system characteristics, user perceptions, and behavioral impacts. Int J Man Mach Stud.

[24] Cook E (2013). Who Uses NHS Direct? Factors That Impact on Telephone Based Healthcare.

[25] Topacan U, Basoglu N, Daim T (2009). Health information service adoption: case of telemedicine. 42nd Hawaii International Conference on System Sciences: 2009; Hawaii, USA.

[26] Walker RH, Johnson LW (2006). Why consumers use and do not use technology-enabled services. J Serv Mark.

[27] NHS Direct (2009). NHS Direct: understanding barriers amongs younger and older people. Qualitative Research.

[28] Kitzinger J (1995). Qualitative research: introducing focus groups. BMJ.

[29] Powell RA, Single HM (1996). Focus groups. Int J Qual Health Care.

[30] Foster J, Dale J, Jessopp L (2001). A qualitative study of older people’s views of out-of-hours services. Br J Gen Pract.

[31] Campbell SM, Rowland MO (1998). Why do people consult the doctor?. Fam Pract.

[32] Neale J, Worrell M, Randhawa G (2009). Barriers to accessing mental health support services: A qualitative study among young South Asian and African Caribbean communities. J Public Ment Health.

[33] Patton MQ (2002). Qualitative Research and Evaluation Methods.

[34] Morgan DL (1998). The Focus Group Guidebook.

[35] Shaw M, Dorling D, Brimblecombe N (1998). Changing the map: health in Britain 1951–91. Sociol Health Illn.

[36] Wohland P, Rees P (2010). Life expectancy variation across England’s local areas by ethnic group in 2001. J Maps.

[37] **Mid-2008 Population Estimates** [www.statistics.gov.uk]

[38] ONS (2006). Life Expectancy at Birth (Years) and Rank Order, by Local Authority in the United Kingdom, With 95% Confidence Limits 1993–1995 and 2003–2005.

[39] Manchester Partnership (2011). Manchester: State of the City Report 2009/2010.

[40] Mendip District Council (2011). Mendip Spatial Patterns: An Analysis of Spatial Variances in Social, Economic and Environmental Factors in Mendip District.

[41] Experian Ltd (2004). Mosaic United Kingdom: The consumer classification for the UK.

[42] Kreuter MW, McClure SM (2004). The role of culture in health communication. Ann Rev Public Health.

[43] Ullah W, Theivendra A, Sood V, Vasireddy A, Maryon-Davis A (2003). Men and older people are less likely use NHS Direct. BMJ.

[44] David OJ (2005). NHS Direct and older people. Age Ageing.

[45] Ritchie J, Spencer L, Bryman A, Burgess R (1994). Qualitative data analysis for applied policy research. Analysing Qualitative Data.

[46] Ritchie J, Spencer L, Bryman A, Burgess RG (1994). Qualitative data analysis for applied policy research. Analyzing Qualitative Data.

[47] Ritchie J, Lewis J (2003). Qualitative research practice: a guide for social science students and researchers.

[48] **How To Contact NHS Direct** [http://webarchive.nationalarchives.gov.uk/20080804123720/direct.gov.uk/en/HealthAndWellBeing/HealthServices/PractitionersAndServices/DG_10036716]

[49] George S (2002). NHS Direct audited: customer satisfaction, but at what price. BMJ.

[50] Flick U (2000). Qualitative enquiries into social representations of health. J Health Psychol.

[51] Virokannas H, Rahkonen M, Luoma I, Sorvari M (2000). The 60-year-old female worker as user of new technology. Int J Ind Ergon.

[52] Millward P: **The‘grey digital divide’: Perception, exclusion and barriers of access to the Internet for older people**. *First Monday* 2003, **8**(7). http://firstmonday.org/ojs/index.php/fm/article/view/1066

[53] Dobransky K, Hargittai E (2006). The disability divide in internet access and use. Information, Communication & Society.

[54] Mollenkopf H, Kaspar R, Guerci A, Consigliere S (2002). Attitudes to technology in old age preconditions for acceptance or rejection. Living in Old Age: Western World and Modernization.

[55] Morris MG, Venkatesh V (2000). Age differences in technology adoption decisions: implications for a changing work force. Pers Soc Psychol.

[56] Mollenkopf H, Charness N, Schaie KW (2003). Assistive technology: potential and preconditions of useful applications. Impact of Technology on Successful Ageing.

[57] Selwyn N, Gorard S, Furlong J, Madden L (2003). Older adults’ use of information and communications technology in everyday life. Ageing Soc.

[58] Tacken M, Marcellini F, Mollenkopf H, Ruoppila I, Szeman Z (2005). Use and acceptance of new technology by older people. Findings of the international MOBILATE survey:‘enhancing mobility in later life’. Gerontechnology.

[59] Parent F, Coppieters Y, Parent M (2001). Information technologies, health, and globalization: anyone excluded?. J Medical Internet Res.

[60] Raynes N, Margiotta P, Lawson J, Pagidas D (2003). The provision of information and advice for older people: what more do they want?. Qual Ageing.

[61] Psoinos M, Hatzidimitriadou E, Butler C, Barn R (2011). Ethnic monitoring in healthcare services in the UK as a mechanism to address health disparities: a narrative review.

[62] Jones R (2003). Making health information accessible to patients. Aslib Proc.

[63] Ali N, Atkin K, Neal R (2006). The role of culture in the general practice consultation process. Ethn Health.

[64] Schouten BC, Meeuwesen L (2006). Cultural differences in medical communication: a review of the literature. Patient Educ Couns.

[65] Bridges J (2008). Listening Makes Sense: Understanding the Experiences of Older People and Relatives Using Urgent Care Services in England.

