# Supplementary material for: Barriers and facilitators to using NHS Direct: a qualitative study of ‘users’ and ‘non-users’
Source: BMC Health Serv Res. 2014 Oct 25;14:487. doi: 10.1186/s12913-014-0487-3 (PMC4220056; doi:10.1186/s12913-014-0487-3)
Supplement: Additional file 1: — An overview of NHS Direct. [file 12913_2014_487_MOESM1_ESM.doc]

# Additional file 1. An overview of NHS Direct

NHS Direct was a health advice and information service application which provided multichannel healthcare in England and Wales, 24 hours a day, and 365 days a year. NHS Direct operated 33 sites, all connected via a single virtual contact center, which offered the 0845 4647 core service which alone answered over 5 million calls a year . The core service essentially offered a patient centred service offering; clinical assessments to support people to care for themselves at home, information on local health services and support organisations, advice on maintaining a healthy lifestyle, information surrounding illnesses, conditions, tests, treatments and operations, medicinal enquiries alongside information regarding health pandemics . Furthermore, NHS Direct also offered commissioned services including; out of hours support for GP’s and dental services, telephone support for patients with long term conditions, pre and post-operative support for patients and finally, remote clinics via telephone .

NHS Direct online, another service provided by NHS Direct also become a popular resource since its inception in December 1999, receiving over 3 million visits every month . The website provided clinically accurate and up to date information set to work in sync with the telephone based service, allowing the public to access clear and straightforward health information anonymously. The website operated many applications which include; NHS Direct online also managed a self-assessment tool (SAT) which around 5 million people a year visited . This service allowed patients to disclose their symptoms online through an algorithm based checklist which provided them self-care advice along with a call back option if needed. There was also specialist advice on issues such as mental health, contraception and pregnancy at work, alongside a General Assessment Tool which provides a symptom checker where patients could use an interactive body map.

# References

1. NHS Direct **NHS Direct: Annual Report & Accounts 2008/9**. In*.* London: The Stationery Office; 2009.

2. NHS Direct: **NHS Direct: National Health Service Trust. Annual Report & Accounts 2009/2010**. In*.*: NHS Direct; 2010.

3. NHS Direct: **Annual Report and Accounts 2009/10**. In*.* London: NHS Direct; 2010.
